# Molecular mechanisms of Kir channel gating

**DOI:** 10.1042/BST20260102

**Published:** 2026-08-13

**Authors:** Arpan Bysack, Hariharasundaram Raghuraman

**Affiliations:** 1 Biophysical Sciences Division, Saha Institute of Nuclear Physics, Kolkata, India; 2 Homi Bhabha National Institute, Training School Complex, Mumbai, India

**Keywords:** Inward-rectifying K+ channel, lipid-dependent gating, small-molecule modulators, structural dynamics, voltage-dependent pore block

## Abstract

Inward-rectifying K^+^ (Kir) channels are ubiquitously present in variety of cells and play an important role in maintaining resting membrane potential and supporting K^+^ homeostasis. They are an important family of K^+^ channels that connects cellular metabolism to membrane excitability, and exhibit complex lipid–protein interaction landscape. Dysfunction of Kir channels is, therefore, associated with multi-factorial diseases and are important drug targets. Recent high-resolution structural dynamics and functional studies of several Kir channels have significantly advanced our understanding of the mechanisms of voltage-dependent pore block and channel gating regulation mediated by lipids, and other modulators. In the present minireview, based on recent knowledge derived from prokaryotic and eukaryotic Kir channels, we highlight the binding sites of the channel for various ligands/modulators, and also provide an emerging model focusing on the structural rearrangements associated with the transition of the channel from the closed/deactivated to open/activated conformation during lipid-dependent gating, which should be broadly applicable to all Kir channels.

## Introduction

In the human genome, ∼80 genes encode potassium (K^+^) channels making them the largest family among ion channels [1]. They assemble as homo- or heterotetramers for K^+^ transport and perform various cellular processes, which include membrane excitability, hormone secretion, and cell volume regulation. Particularly, the inward-rectifying K^+^ (Kir) channel family has seven members (Kir1.x–Kir7.x), which are encoded by 16 genes [2–4]. Based on the sequence homology, Kir channels are mainly divided into four functional classes namely the classical Kir (Kir2.x), G-protein coupled Kir (Kir3.x), ATP-sensitive K^+^ channels (Kir6.x), and K^+^ transport (Kir1.x, Kir4.x, Kir5.x, Kir7.x) channels. Kir channels are present in almost all cells and, unlike voltage-gated K^+^ (K_v_) channels, their function is characterized by the ability to conduct K^+^ ions more easily into the cell at hyperpolarizing potential rather than out of it at depolarizing potential. Therefore, the ‘inward’ (formerly ‘anomalous’) rectification is the phenomenon whereby K^+^ conductance is significantly reduced at voltages positive to its reversal potential [5]. This rectification property of Kir channels is a result of pore blockage at depolarizing potentials mediated by intracellular polyamines such as spermine and putrescine and Mg^2+^ ions [2,6,7]. Importantly, this rectification process has two significant consequences—firstly, high K^+^ conductance at negative potentials ensures that cells maintain a stable resting membrane potential upon hyperpolarizing factors. Secondly, very low K^+^ conductance at positive potentials prevents excessive outward K^+^ flow during the action potential in excitable cells such as cardiac myocytes enabling stable cardiac rhythm. Depending on their sensitivity to intracellular blockers and rectification properties, Kir channels are categorized as ‘strong’, ‘intermediate’, or ‘weak’ rectifiers [2,3,8].

Apart from voltage-dependent conductance changes of Kir channels, membrane lipids, particularly phosphatidylinositol-4,5-bisphosphate (PIP_2_) and cholesterol, critically regulate the activation and deactivation mechanisms thereby making these channels undergo lipid-dependent gating. In fact, certain loss-of-function mutations in the Kir channel family, which impairs PIP_2_-dependent activation gating, are directly associated with numerous disorders [9] such as Bartter’s syndrome type 2 (Kir1.1), Andersen’s syndrome/long QT syndrome 7 (Kir 2.1), long QT syndrome 13 (Kir3.4), EAST/SeSAME syndrome (Kir4.1), hypokalemic tubulopathy and deafness (Kir5.1), Cantu syndrome (Kir6.1), hyperinsulinemic hypoglycemia familial 2 (Kir6.2), Leber congenital amaurosis, and snowflake vitreoretinal degeneration (Kir7.1). Due to pathophysiological consequences of the loss-of-function mutations, Kir channels are, therefore, promising drug targets. Several excellent reviews are available in the literature that extensively discuss the Kir channel family, physiology, their structures, pharmacology, and mechanisms of gating [2,3,7,9–17]. In the present review, we give an overview of Kir channels and discuss various modulators that affect the gating. Further, based on the recent structural data derived from prokaryotic and eukaryotic Kir channels, we provide an emerging model that incorporates key structural features when the channel shuttles from the closed/deactivated to open/activated conformation during lipid-dependent gating.

## Structures of Kir channels

**Figure 1:**
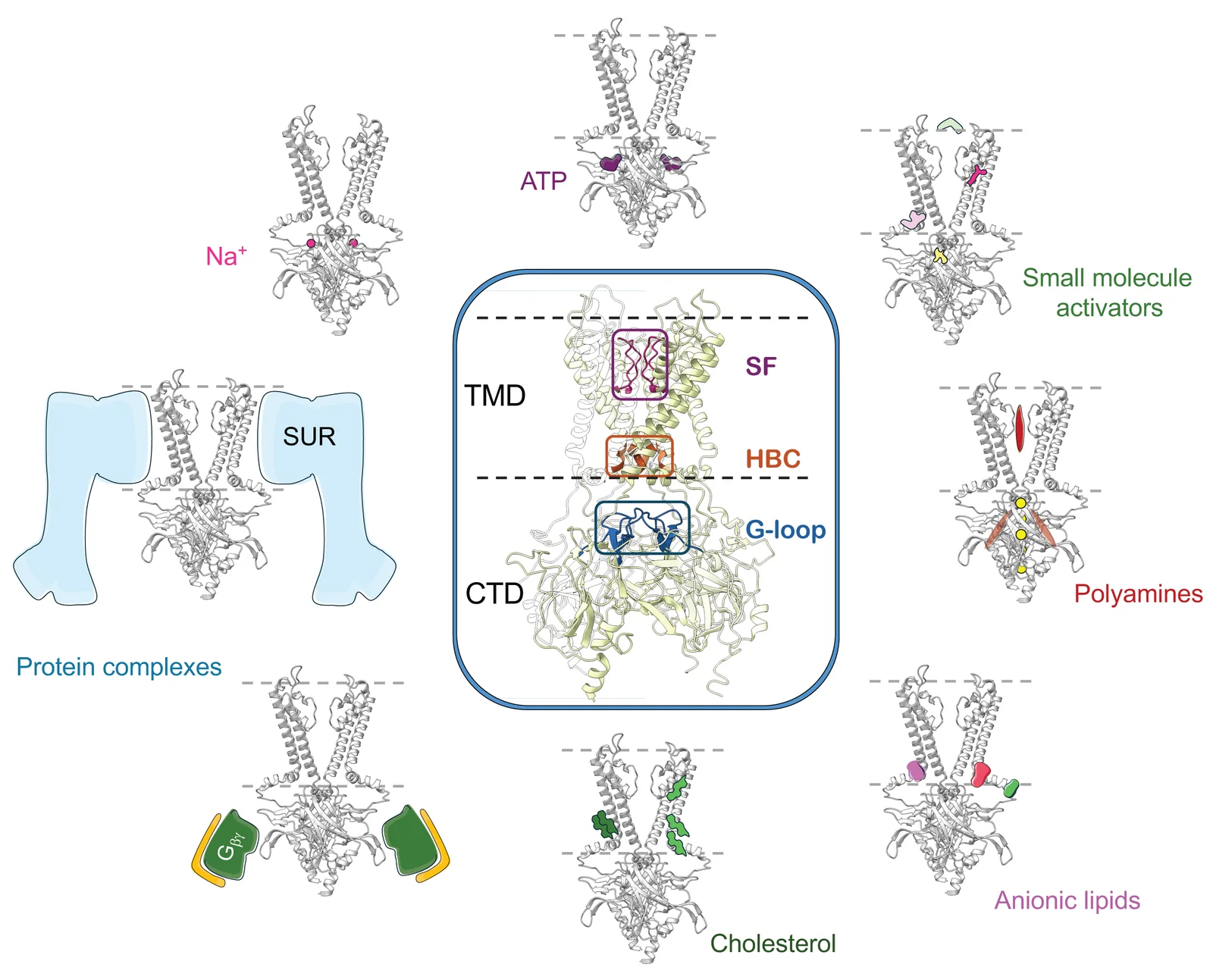
Kir channel is regulated by diverse modulators Cartoon representation of the closed-state cryo-EM structure of the homotetrameric Kir2.1 channel (PDB: 7ZDZ), showing the TMD and cytoplasmic domain (CTD). Three gates namely the selectivity filter (SF), HBC/inner gate, and the G-loop gate involved in channel gating and K^+^ permeation are highlighted. Schematic representation of probable binding sites of key modulators of Kir channel (only diagonal subunits are shown in gray) ranging from a small Na^+^ ion to large protein complexes is shown. The depicted small-molecule activators include flecainide (yellow), naringin (light green), ML297 (magenta), and ivermectin (light pink), which bind to different regions of the channel. In case of polyamines, binding sites associated with the weak (pale red) and strong (dark red) voltage-dependent rectification are depicted along with the K^+^ ions (yellow spheres) at the CTD. Anionic lipids PIP_2_ (pink) and PG (red) at the primary lipid-binding site proximal to the HBC and slide helix, whereas the anionic lipid PS (green) at the secondary lipid-binding site near the N-terminus of the slide helix. Cholesterol as a monomer (light green) or a dimer (dark green), is depicted to interact with different regions of TMD. Interactions of regulatory proteins like G_βγ_ (green–yellow) and SUR (cyan) to form protein complexes are shown. Arbitrary membrane boundaries are indicated by dotted lines. See text for details.

Over the last two decades, several high-resolution structures of prokaryotic and eukaryotic Kir channels have been determined, which reveal a highly conserved architecture [18–28]. It should be noted that, to date, no high-resolution open state structure(s) of full-length wild-type Kir channel have been obtained, and the available open state structures are with gating-facilitating mutations (see Supplementary Table S1). The functional unit of Kir channels is homo- or heterotetramer with four-fold symmetry about a central pore consisting of α-helical transmembrane domain (TMD)—each subunit has two α-helices M1 and M2—and a cytoplasmic domain (CTD) predominantly composed of β-sheets (see Figure 1). From the pore-domain perspective, Kir channels share most of the structural features with KcsA K^+^ channel [29–31], i.e., an outer vestibule, selectivity filter, pore helix, and an N-terminal amphipathic helix, which is termed the ‘slide helix’ or ‘interfacial helix’ in Kir channels. There are three ‘gates’ that govern the K^+^ permeation through Kir channels: (i) selectivity filter; (ii) helix-bundle crossing (HBC) at the intracellular side of TMD; and (iii) G-loop at the top of CTD. Figure 1 shows the basic architecture of Kir channels that highlights the TMD, CTD, and the three functionally relevant ‘gates’ along with the probable binding sites of key modulators, which range from Na^+^ ions to lipids to large protein complexes, that critically regulate the voltage-dependent conductance change and lipid-dependent gating of Kir channels.

## Modulators of Kir channel gating

Kir channels are arguably the most well-studied system, whose subtype is sensitive to different and diverse modulators that regulate channel gating. We summarize the effects of various modulators that range from as small as sodium ions to protein complexes on Kir channel function.

### Voltage-dependent binding of polyamines

As mentioned above, the binding of intracellular polycationic metabolites (i.e., polyamines particularly spermine and spermidine) to Kir channels at positive membrane potentials typically block K^+^ permeation through open channels is a well-studied phenomenon and forms the basis of strong inward rectification [5,7]. However, structural evidence for the mechanism of polyamine block is lacking as the high-resolution structure of Kir channel in complex with polyamines is still elusive. A recent study, employing a very long-timescale MD simulation (∼5 milliseconds) and the G178R mutant of the PIP_2_-bound chicken Kir2.2 with a non-conducting pore as the template, shows that significant conformational changes take place at the HBC and the CTD when the channel transitions to a stably open, ion conducting pore [32]. The simulation study clearly shows that single file movement of K^+^ ions does not take place below the constricted G-loop region, clearly not favoring the previously proposed ‘extended pore filing’ model [7] for the polyamine block. However, this study does not address the role of the structurally resolved narrow constriction (‘gasket’) in the cytoplasmic pore in voltage-dependent block of polyamines as seen in the structure of the isolated cytoplasmic portion of Kir3.1 with S225E mutation [33]. Interestingly, spermine expels 5–6 K^+^ ions above the G-loop region, which is consistent with the notion that strongly coupled single file movement of K^+^ ions above the G-loop are responsible for the strong voltage dependence of polyamine blocking [7]. It appears that, depending on the applied computational voltages, spermine binds to different regions of the Kir channel, and the ‘cavity trapping’ model of rectification [5], in which the spermine binds predominantly at inner cavity and the selectivity filter seems plausible.

### Lipids

Biological membranes define cellular boundaries and regulate the selective passage of molecules across them [34]. The distinct classes of lipids and sterols in biomembranes actively regulate protein structure and function in general, and ion channels in particular, through dynamic lipid–protein interactions that supports diverse physiological processes [35–40]. Among ion channels, Kir channel family represents a well-established example of complex lipid-dependent regulation of function [8,10,41,42]. KirBac1.1 is the extensively studied prokaryotic homolog for understanding lipid-dependent gating mechanism of Kir channels [43–50]. Like its eukaryotic counterpart, the tetrameric KirBac1.1 lacks a canonical voltage-sensor domain and is regulated by different lipids mainly at the transmembrane–cytoplasmic interface. Notably, KirBac1.1 shares >50% sequence homology with mammalian Kir2 channels [51].

The activation of all eukaryotic Kir channels essentially requires binding of PIP_2_ at the intracellular leaflet [10,52]. In eukaryotes, the negatively charged PIP_2_ is the most abundant phosphatidylinositol phosphate lipid species with a net charge of −3 to −4 at physiological pH, and is crucial for cellular signaling [53], and constitutes ∼2% of the total lipid content in cellular membranes [54]. Hansen and coworkers [21] provided the first high-resolution crystal structure of chicken Kir2.2 bound to activating ligand PIP_2_. Although this structure has advanced our knowledge on the structural basis of PIP_2_ activation of Kir 2.2 channels, the HBC gate was too narrow to be called the open state. The first acceptable open structure of Kir channel has been determined using the gain-of-function mutations harboring Kir6.2 complexed with SUR1 [7,24]. Importantly, the PIP_2_ binding mediated functional effects of prokaryotic and eukaryotic Kir channels are complex. While the eukaryotic Kir channels are activated by PIP_2_, it inhibits the K^+^ transport activity of the prokaryotic Kir (KirBac1.1) channels [47,55,56]. This discrepancy could be due to the presence of longer N- and C-terminal linkers that contain key residues for PIP_2_-mediated activation in eukaryotic Kir channels [57]. This shows that the mere presence of anionic lipids in membranes is not sufficient to activate the channel, rather highlights the importance of specific lipid–protein interactions. This is obvious in case of KirBac1.1 where anionic phospholipids such as phosphatidylglycerol (PG) and cardiolipin stabilize the active/open state [46,47,49,50], which is contrary to the functional effects of PIP_2_ [55,56]. Further, in chicken Kir2.2, small headgroup anionic lipid, pyrophosphatidic acid, has been shown to be incompetent in activating the channel in the absence of PIP_2_, probably due to its inability to dock the CTD with the TMD [21,58]. Apart from the primary lipid binding site where PIP_2_ binds, MD simulation studies have identified putative secondary phospholipid binding sites in Kir2.1 and Kir2.2 channels, where phosphatidylserine (PS) binds [41,42]. Overall, these studies underline the need to consider the role of multiple lipid species in channel–membrane interactions.

Cholesterol is a predominant sterol in mammalian cell plasma membrane (∼35–45 mol%) and is severely depleted in intracellular membranes [38,59]. Importantly, cholesterol is a major regulator of the function of different classes of membrane proteins like ion channels, transporters and G-protein coupled receptors through direct molecular interactions and/or changes in the membrane bilayer properties [60–62]. In particular, cholesterol inhibits the Kir channel activity in most cases [16,28,63,64] even the prokaryotic KirBac1.1 channel [65,66]. However, some GIRK (Kir3.x) channels get activated by cholesterol [63,67,68]. This illustrates the complex nature of Kir channel regulation by membrane cholesterol. Interestingly, cholesterol inhibition of channel activity is substantial even in the presence of activating anionic lipids PIP_2_ and PG in Kir [28] and KirBac1.1 [65,66], respectively. The cholesterol-induced suppression of Kir channels is probably mediated by decoupling at the inter-subunit interfaces, i.e., it reduces the likelihood of contact between specific regions of the CTD and TMD [28,64]. Despite different lipids exert varying regulation of Kir channel activity, it appears that the primary site responsible for lipid-dependent gating is near the HBC at the cytoplasmic side consisting of residues surrounding transmembrane (TM) helices and the slide helix [21,48,65,69,70].

### Small-molecule activators

The development of selective pharmacological modulators for K^+^ channels, and Kir subtypes in particular, remains intrinsically challenging owing to the conserved architecture and shared structural features of the K^+^ channel superfamily. Nonetheless, because many loss-of-function Kir variants cause genetic disorders that disrupt PIP_2_-dependent activation gating, subtype-selective Kir activators represent an attractive therapeutic strategy for channelopathies associated with impaired channel opening [9,71]. Despite this rationale, Kir activator discovery remains markedly less advanced than inhibitor development, and most newly identified Kir-directed ligands are blockers [72,73].

Within the Kir2.x subfamily, flecainide, propafenone, and timolol activate Kir2.1 through interaction with cytoplasmic-domain residues that reduce polyamine affinity and thereby enhance inward current [6]. Medicinal chemistry optimization subsequently yielded GPV0057, a more selective Kir2.1 activator with reduced off-target activity. Additional Kir2 modulators include pregnenolone sulphate and aminophylline, which exerts mixed effects on inward rectifier currents [9]. Kir3.x (GIRK) channels constitute the most mature area of Kir activator pharmacology, with compounds including naringin, ML297, VU0810464, GAT1508, GiGA1, ivermectin, and the GIRK4-selective modulator 3hi2one-G4 collectively establishing the tractability of subtype-selective GIRK activation [74]. Apart from chemical modulators, in GIRK channels, intracellular Na^+^ functions as an endogenous allosteric activator by binding within the CTD loops of GIRK2 and GIRK4 subunits, where coordination by a conserved Asp residue enhances channel affinity for PIP_2_ [16,75]. Beyond Kir2 and Kir3, luteolin potentiate Kir4.1 channels [76], whereas CL-705G selectively activates Kir6.2/SUR2A channels [77]. However, translational progress remains constrained by the PIP_2_-dependence of most activators, incomplete metabolic and pharmacokinetic characterization, limited structural definition of ligand-binding sites, and the difficulty of achieving tissue-selective modulation across broadly expressed Kir subtypes [9].

### Protein complexes

The complex nature of the regulation of PIP_2_-mediated Kir channel activation in membranes also involves protein-protein interactions with the interdependence of multiple ligands, which has been established in GIRK (Kir3.x) channels [16,78] and K_ATP_ channels [15,24,25,70]. The uniqueness of GIRK channels is that robust channel opening requires the presence of both PIP_2_ and G_βγ_ as seen in planar bilayer electrophysiology experiments with neuronal GIRK2 [78]. Interestingly, GIRK channels are regulated by G_βγ_ subunits associated with ‘inhibitory’ Gα_i/o_ and not by those associated with ‘stimulatory’ Gα_s_, and it should be noted that attempts to purify a stable GIRK-G_βγ_ protein complex in detergent micelles were unsuccessful [79]. Interestingly, Na^+^ increases GIRK2 channel opening in the presence of G_βγ_ and PIP_2_, although Na^+^ alone is not sufficient for the channel activation making it as the modulator of effects of other ligands [78]. G_βγ_ allosterically promotes PIP_2_-dependent gating stabilizing the open state through distinct effects on the HBC and cytoplasmic G-loop gates, respectively [16,75]. The structures of GIRK channels show that, in the absence of PIP_2_, the channel probably exists in the equilibrium between ‘extended’ and ‘docked’ conformation [23,69] much like the classical human Kir2 channels [26]. Binding of PIP_2_ to channel predominantly stabilizes the ‘docked’ conformation to which G_βγ_ binds. Basically, PIP_2_-induced conformational changes open the pore in Kir2.2 channels whereas it prepares the GIRK channels for the activation by G_βγ_ [23].

The ATP-sensitive K^+^ (K_ATP_) channels are hetero-octameric complexes of four Kir 6.1/6.2 channels and four regulatory sulfonylurea receptors (SUR1, SUR2A, or SUR2B) from the ABC transporter family [15]. These channels are regulated by intracellular ATP and ADP. The regulation of channel activity involves both Kir and SUR subunits. While the non-hydrolytic binding of ATP to Kir6 inhibits the channel activity, as mentioned before, the PIP_2_ binding to Kir6 stimulates the channel activity leading to a competitive gating by ATP and PIP_2_. Interestingly, SUR enhances the channel sensitivity to both these ligands by ∼10-fold [15,24,80]. Recent cryo-EM structure of the K_ATP_ channel, with Kir6.2 in the open conformation, shows two PIP_2_ binding sites: one corresponds to the canonical PIP_2_ binding site observed in all Kir channels, and the other binding site (non-canonical) located at the interface of the complex in which the PIP_2_ is coordinated by both Kir6.2 and SUR1 subunits [70].

## Structural rearrangements during lipid-dependent gating of Kir channels

**Figure 2:**
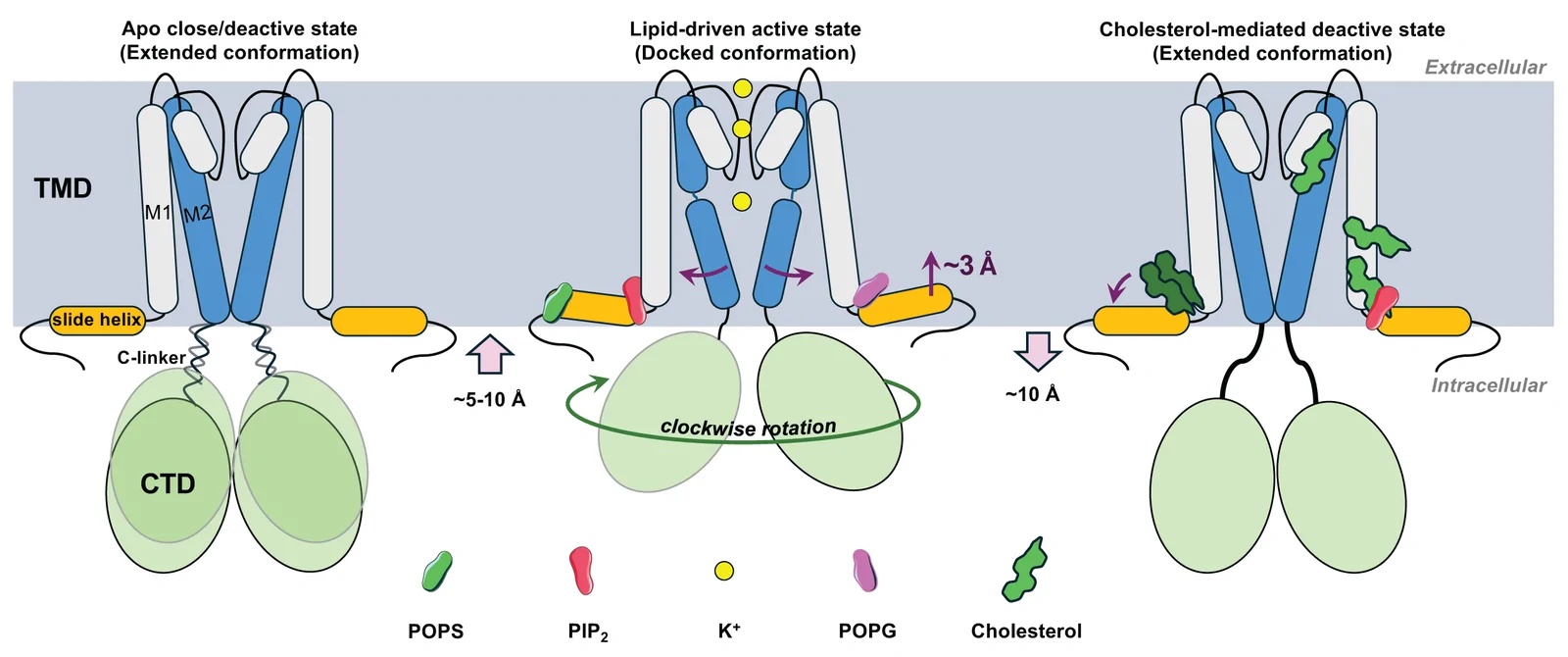
Lipid-dependent gating mechanisms in Kir channels Shown are the schematic representation of the diagonal subunits of a Kir channel highlighting the outer (M1) and inner (M2) TM helices, TMD (gray shaded), the slide helix (yellow), C-linkers, and the CTD (green). *Left*: In the apo closed state, disengagement of the CTD from the TMD renders the connecting C-linkers disordered and behave like a ‘spring’ (depicted as wiggly lines), allowing the CTD to sample ‘extended’ and ‘docked’ conformations and resulting in substantial conformational heterogeneity. Middle: Depending on Kir or KirBac channels, binding of anionic lipids PIP_2_ or PG at the primary lipid binding site (proximal to the HBC) and the anionic lipid PS at the secondary lipid-binding site (near the N-terminus of the slide helix), results in ‘kink’ at the conserved Gly residue and inward rotation of M2 helices and a ‘twist-to-dock’ conformational transition of the CTD, which is characterized by the clockwise rotation of the CTD and an upward displacement of ∼5–10 Å toward the TMD thereby stabilizing the active state (‘docked’ conformation) allowing efficient K^+^ (yellow spheres) permeation. During this transition to the active state, the slide helix penetrates deeper within the membrane interface by 3 Å. Right: Cholesterol binding at the TMD, either as monomer (light green) or dimer (dark green), restricts M2 hinge movement along with C-linkers (depicted as thick black lines) and stabilizes the CTD in an extended conformation (∼10 Å downward displacement from the TMD), thereby inhibiting lipid-induced channel activation even in the presence of activating gating ligand PIP_2_ or PG.

Numerous high-resolution crystal and cryo-EM structures of prokaryotic and eukaryotic Kir channels in apo- and bound to various modulators are now available (see Supplementary Table S1), which provides detailed mechanistic insights to decades of functional data to understand Kir channel gating mechanisms. Kir channel activation gating typically involves the conformational changes that couples (i.e., docks) the TMD and CTD via the slide helix and C-linkers to open the gates for the eventual K^+^ permeation. In the apo closed/deactivated state of a Kir channel, due to its disengagement with the TMD, the disordered connecting linkers between the TMD and CTD act as a spring to shuttle the CTD between the ‘extended’ and ‘docked’ conformation [26], i.e., the CTD exhibits high degree of conformational heterogeneity (Figure 2, left). Particularly, the anionic lipid PIP_2_ binding at the interface between the TMD and CTD in all four subunits (with conserved Arg and Lys residues) involves varied amounts of clockwise rotation of the CTD relative to the TMD of the same subunit in both KirBac and Kir channels despite the contrasting functional outcome [52]. For instance, PIP_2_-bound structures of Kir channels show that the clockwise rotation of CTD is reported to be ∼13º in Kir 6.2 [25], 31º–38º in Kir 3.2 [23,81], and 45º in Kir7.1 [28]. Further, MD simulation studies report a value of 80º rotation for Kir2.2 [52]. In Kir, the entire CTD translates to 5–10 Å toward the TMD during PIP_2_ activation, i.e., the CTD transitions from the ‘extended’ to ‘docked’ conformation that leads to HBC gate opening (Figure 2, middle). Although Kir channels share similar architecture with KcsA, one notable structural feature relevant for gating and K^+^ permeation is the absence of large openings of the HBC. For instance, the full-length and the CTD truncated version of KcsA opens up to 21 Å [82] and 32 Å [83], respectively, compared to the distance of 12 Å (C_a_–C_a_ distances at Thr112) in the closed state. None of the reported high-resolution structures of Kir channels display such a large degree of HBC openings even under conditions of channel activation (see Supplementary Table S1). This might probably suggest that hydrophobic interactions, which maintain the closure of the HBC gate are very strong, and the bulky nature of CTD in Kir channels might also impose structural constraints for a large HBC opening. However, it is also possible that these structures are only determined in detergent micelles and not in physiologically relevant membranes, and therefore they might not represent the true constitutively open state.

In contrast to eukaryotic Kir channels, KirBac is activated by anionic lipids PG [46–50] and cardiolipin [47]. Key mechanistic insights into Kir channel gating have been derived from bacterial homologues, which established the CTDs ‘twist-to-open’ mechanism for Kir channel gating [20,84], and the pore opening involves bending and twisting in M2 helix at the conserved glycine residue (acts as an ‘hinge’) thereby dilating the HBC gate [84]. Similar conformational transitions were observed in KirBac1.1 during PG-dependent activation in membranes using solid-state NMR spectroscopy [47–49]. It is well known that the slide helix, residing at the cytoplasmic membrane interface of Kir channels, functions as a critical coupling hub between the TM pore and CTD, transmitting conformational changes from ligand binding sites. Despite some confusion regarding the slide helix position, recent work clearly demonstrates that the functionally pertinent slide helix resides at the membrane interface irrespective of the functional states [50,85]. Further, the slide helix position in the membrane might act as a ‘conformational switch’ in regulating Kir channel function [50,66]. Although upward CTD translation remains incompletely resolved in prokaryotic Kir channels, the PG-driven activation of KirBac1.1 involves an ∼3 Å upward displacement (i.e., deeper membrane partitioning) of the slide helix that is associated with altered conformational heterogeneity [50], which is consistent with the earlier single-molecule FRET results showing that the slide helix undergoes marked conformational fluctuations [43]. Overall, both in bacterial and eukaryotic channel, the activation mechanism involves rigid body rotation of the CTD domain and upward translational motion (docking like a screw) (Figure 2, middle).

Conversely, cholesterol mostly stabilizes the close/deactivated Kir conformations (see Figure 2, right) and is associated with ∼5–10 Å downward CTD displacement in most Kir channels [23,28], although dual-nature of cholesterol regulation is reported in GIRK channels [81,86] and needs further exploration [16]. Docking experiments predict that, in both KirBac1.1 and Kir2.2, cholesterol is wedged between TM1 and TM2, and its hydroxyl group facing the cytoplasmic side of the membrane and interacts with the slide helix within the same subunit [87,88]. Complex nature of cholesterol-KirBac1.1 has also been proposed using solid-state NMR and computational approaches in which cholesterol binds to channel as dimer with two poses (intra-subunit and inter-subunit) near the HBC involving the slide helix and TM helices [65]. This study is one of the first examples of a specific interaction of a cholesterol dimer that regulates the ion channel activity, and probably gives a new dimension for the role of cholesterol in membrane protein function. Overall, these studies point to the fact that the role of slide helix in cholesterol recognition is undisputed. Recent Kir7.1 structures show that cholesterol stabilizes the extended closed/deactive conformation even in the presence of activating ligand PIP_2_ [28]. It appears that cholesterol binding to Kir channels, whose CTDs are already in the docked conformation due to PIP_2_-induced activation, locks the TM helices at a fixed angle and straightens the outer M1 helix, which might destabilize the inter-subunit contacts between the CTD and TMD resulting in the disengaged extended conformation of the CTD [28,64,89].

## Conclusions

As mentioned, decades of functional data and advances in structural biology, particularly in cryo-EM structure determination, have enabled a better understanding of the gating mechanisms of Kir channels. The common theme arises is the clockwise rotation of CTD and its docking to the TMD during Kir channel gating. In other words, the channel assumes the ‘extended’ and ‘docked’ conformations of CTD with varying degrees of CTD rotation in close/deactive and active conformations, respectively. How much of minimum CTD rotation is required to establish the inter-domain connections in PIP_2_-mediated activation remains obscure. From the existing literature, it also remains unclear why PIP_2_ alone can robustly activate Kir2 channels, whereas it fails to activate GIRK (Kir3.x) channels by itself and requires G_βγ_ for the robust activation. Further, with respect to cholesterol-channel structural studies, due to its relatively high solubility in maltoside detergents [90], cholesteryl hemisuccinate (CHS) is generally used as a surrogate for cholesterol. Unfortunately, it is known that CHS does not always bind to membrane proteins in the same way as cholesterol [91,92]. More studies are needed to understand the molecular basis of the dual-nature of cholesterol in Kir channel regulation.

Although numerous high-resolution structures of Kir channels bound to various modulators are now available, the structures were determined in micellar environment and not in the physiologically relevant membrane environment. Since membrane shapes protein structure and vice versa [93], solving the structures of Kir channels directly in lipid bilayer will help us to understand the intricate details of general and specific lipid–protein interactions as shown recently for other channels [94–96]. Importantly, recent studies show that the structural dynamics of several membrane proteins, particularly KirBac1.1 [85], is significantly altered in micelles and membranes [97–102]. In addition, although the structural insights provide the mechanistic details of the gating mechanism of Kir channels, the structural dynamics associated it are not well understood. Therefore, the structural data should be complemented by the sophisticated spectroscopic approaches performed in proteoliposomes. For instance, distance measurements in membrane set-up [43,103,104] will provide greater insights into the nature of HBC gate opening and tilting of helices during gating, and two-dimensional infrared spectroscopy can offer the ion occupancy states and the dynamics of the selectivity filter gate [29,105].

## Perspectives

- Kir channels maintain the resting membrane potential of the cell, and multiple human diseases are linked to mutations in Kir genes, making Kir channels potential drug targets.
- The function of Kir channels is regulated by diverse modulators and specific lipid–protein interactions by multiple lipid species, and display a complex channel–membrane interaction landscape. MD simulation with previously unattainable timescales (∼5 milliseconds) provides the molecular visualization of channel block by spermine.
- Structural data in micelles reveal that CTD of Kir channels in Apo closed state is in ‘extended’ conformation, whereas in PIP_2_-mediated active state, the CTD adopts a ‘docked’ conformation (up by 5-10 Å) with TMD. This requires validation from sophisticated spectroscopic approaches in membranes.

## Supplementary Material

Supplementary Table S1

## Data Availability

All data are contained within the manuscript and the Supplementary Table S1.

## Open Access

This article has been published open access under our Subscribe to Open programme, made possible through the support of our subscribing institutions, learn more here: https://portlandpress.com/pages/open_access_options_and_prices#conditional.

## Abbreviations

CHS: cholesteryl hemisuccinate
CTD: cytoplasmic domain
HBC: helix-bundle crossing
Kir: inward-rectifying K^+^
PG: phosphatidylglycerol
PIP_2_: phosphatidylinositol-4,5-bisphosphate
PS: phosphatidylserine
TM: transmembrane
TMD: transmembrane domain
