# Supplementary material for: Molecular mechanisms of Kir channel gating
Source: Biochem Soc Trans. 2026 Aug 13;54(8):1095–106. doi: 10.1042/BST20260102 (PMC13488966; doi:10.1042/BST20260102)
Supplement: Supplementary file 1 [file BST-2026-0102C_supp.pdf]

Supplementary Table 1: High resolution structures# of prokaryotic and eukaryotic inward rectifying K^+ channels

Structures of prokaryotic Kir channels											
**Protein**	**Organism**	**Ligand**	**PDB ID**	**Resolution**	**Mutation**	**Membrane mimetic**	**Technique used**	**Distance between C_α-C_α of the residue$^\$$**		**Protein structure**	**Ref.**
KirBac1.1	*Burkholderia pseudomallei*	--	1P7B	3.65 Å	--	Cymal-5	X-ray diffraction	A147	14.18	Full-length	[18]
		--	2WLL	3.65 Å	--		Refined		14.34	Full-length	[20]
		--	7SWJ	--	I131C	DM	Refined (SS-NMR)		14.83	Full-length	[106]
KirBac3.1	*Magnetospirillum magnetotacticum*	--	2WLJ	2.60 Å	--	--	Refined	A133	15.24	Full-length	[20]
			2WLK	2.80 Å	--				15.21		
			2WLI	3.09 Å	--	Tri-DM, LDAO	X-ray diffraction		--		
			2WLM	3.61 Å	--	Tri-DM, LDAO			15.58		
			2WLN	3.44 Å	--	Tri-DM, LDAO			15.70		
			2WLH	3.28 Å	--	Tri-DM, LDAO			15.53		
			2X6A	3.10 Å	Q170A	FC-11-6U; NM			15.01		
		Ba^{2+}	2X6B	3.30 Å	Q170A	FC-11-6U; NM			15.52		
		--	2X6C	2.70 Å	Q170A	FC-11-6U; NM			15.51		
		--	3ZRS	3.05 Å	S129R	HEGA-10	X-ray diffraction		17.07	Full-length	[84]
		--	4LP8	2.46 Å	S129R/S205L	HEGA-10	X-ray diffraction		17.15	Full-length	[107]
		--	6O9U	2.00 Å	--	DDM	X-ray diffraction		14.90	Full-length	[108]
		--	6O9V	3.09 Å	S129-F135C	LDAO			15.44		
		--	7N9L	2.80 Å	W46R	Tri-DM	X-ray diffraction		15.76	Full-length	[109]
		--	7N9K	2.40 Å	C71S-C262S	DDM	X-ray diffraction		15.84	Full-length	[110]
		--	7ADI	2.72 Å	L124M				15.47		

Structures of eukaryotic Kir channels

Protein	Organism	Ligand	PDB ID	Resolution	Mutation	Membrane mimetic	Technique	Distance between C_α-C_α of the residue		Protein structure	Ref.
Kir2.2	*Gallus gallus*	--	3JYC	3.11 Å	--	DM	X-ray diffraction	A182	20.73	Full-length	[19]
		PIP_2	3SPI	3.31 Å	--	DM	X-ray diffraction		14.88	Full-length	[21]
		PPA	3SPC	2.45 Å	--				15.79		
		PIP_2	3SPH	3.00 Å	I223L				14.71		
		--	3SPJ	3.31 Å	I223L				16.46		
		PIP_2	3SPG	2.61 Å	R186A				14.76		
		--	5KUK	2.00 Å	K62W	DM	X-ray diffraction		15.18	Full-length	[111]
		PIP_2	5KUM	2.80 Å	K62W				15.23		
		--	6M86	3.60 Å	K62W - G178D	DM	X-ray diffraction		15.72	Full-length	[22]
		PIP_2	6M84	2.81 Å	K62W - G178D				15.45		
Kir3.1	*Mus musculus*		1N9P	1.80 Å	--	Not applicable	X-ray diffraction	--	--	Cytoplasm ic Domain	[112]
Kir3.1-KirBac1.1 chimera	*Mus musculus/ Burkholderia xenovornas*		2QKS	2.20 Å	--	NG	X-ray diffraction	--	--	Full-length chimera	[113]
Kir3.1	*Mus musculus*		3K6N	2.00 Å	--	Not applicable	X-ray diffraction	--	--	Cytoplasm ic Domain	[33]
Kir3.2	*Mus musculus*		3SYO*	3.54 Å	--	DM	X-ray diffraction	V193	16.17	Full-length	[69]
		PIP_2	3SYA	3.00 Å	--				16.31		
			3SYC	3.40 Å	D228				16.12		
			3SYP	3.10 Å	R201A				16.17		
		PIP_2	3SYQ	3.44 Å	R201A				18.54		
		βγ subunit (human)	4KFM	3.45 Å	--	DDM	X-ray diffraction		16.41	Full-length	[79]
		--	6XIS	3.90 Å	--	DDM; FFC-8	cryo-EM		15.37	Full-length	[23]
		PIP_2	6XIT	3.30 Å	--	DM			16.28		

Kir4.1	*Rattus norvegicus*	PIP_2	8I5M	2.85 Å	--	LMNG	cryo-EM	A167	17.35	Full-length	[114]
		PIP_2 and Lys05	8I5N	2.85 Å	--				16.95		
Kir6.2/ SUR1	*Rattus norvegicus*	ATP, GBC	6BAA	3.63 Å	--	Digitonin	Cryo-EM	M169	14.869	Full-length	[115]
	Rattus norvegicus	ATP, Repaglinide	7TYS	3.41 Å	--	--	cryo-EM reprocessing	M169	14.76	Full-length	[25]
			7TYT	3.60 Å	--	--			15.60		
			7U1Q	3.90 Å	--	--			14.81		
			7U1S	3.80 Å	--	--			14.87		
	Rattus norvegicus	PIP_2	8TI2	3.28 Å	Q52R	Digitonin	cryo-EM	M169	16.89	Full-length	[70]
			8TI1	2.90 Å					16.89		
	Mus musculus	Repaglinide	6JB1	3.30 Å	--	Digitonin	cryo-EM	M169	13.669	Full-length	[116]
	Mus musculus	--	7W4O	2.96 Å	H175K	Digitonin	cryo-EM	M169	16.41	Full-length	[117]
			7W4P	3.19 Å					15.35		
	Homo sapiens	ATP, ADP	6C3O	3.90 Å	--	Amphipol (PMALC8)	cryo-EM	M169	14.88	Full-length	[118]
	Homo sapiens	ATP, ADP	7S5T	3.10 Å	C166S-G334D	GDN	cryo-EM	M169	16.00	Full-length	[24]
			7S5X	3.70 Å					16.51		
			7S5Y	3.90 Å					17.089		
			7S5Z	3.90 Å					16.304		
			7S60	3.70 Å					16.46		
			7S61	4.00 Å					16.01		
Kir6.1/ SUR2B	*Rattus norvegicus*	ATP, Glibenclamide	7MIT	3.40 Å	--	Digitonin	cryo-EM	M179	14.16	Full-Length	[119]
			7MJO	4.00 Å					14.28		
Kir7.1	*Homo sapiens*	Cholesterol, PIP2	9PR5	3.30 Å	--	GDN	cryo-EM	A158	14.87	Full-length	[28]
		PIP2	9PR6	3.90 Å	--				16.26		
		17-OHPC	9PR7	4.00 Å	--				14.77		

Abbreviations: DM: n-decyl-β-D-maltopyranoside; DDM: *n*-dodecyl-β-D-maltopyranoside; FFC8: fluorinated Fos-Choline-8, (1H, 1H, 2H, 2H-perfluorooctyl)phosphocholine; NG: n-nonyl-β-D-glucopyranoside; Cymal-5: 5-cyclohexyl-1-pentyl-β-D-maltoside; LDAO: N,N-dimethyldodecylamine N-oxide; Tri-DM: n-tridecyl-β-D-maltoside; HEGA-10: decanoyl-N-hydroxyethylglucamide, ENT 17OHPC: 17-hydroxyprogesterone caproate

[#] Only structures whose resolution is ≤ 4 Å are tabulated.
[$]calculated values from the corresponding residues in the PDB, compared to T112 in KcsA
[*]First complete structure of a G-protein-gated K^+-selective channel.

Supplementary References

106 Amani, R., Schwieters, C.D., Borcik, C.G., Eason, I.R., Han, R., Harding, B.D. et al. (2021) Water accessibility refinement of the extended structure of KirBac1.1 in the closed state. *Front. Mol. Biosci.* **8**, 772855 https://doi.org/10.3389/fmolb.2021.772855

107 Zubcevic, L., Bavro, V.N., Muniz, J.R., Schmidt, M.R., Wang, S., De Zorzi, R. et al. (2014) Control of KirBac3.1 potassium channel gating at the interface between cytoplasmic domains. *J. Biol. Chem.* **289**, 143-151 https://doi.org/10.1074/jbc.M113.501833

108 Black, K.A., He, S., Jin, R., Miller, D.M., Bolla, J.R., Clarke, O.B., et al. (2020) A constricted opening in Kir channels does not impede potassium conduction. *Nat. Commun.* **11**, 3024 https://doi.org/10.1038/s41467-020-16842-0

109 Fagnen, C., Bannwarth, L., Oubella, I., Zuniga, D., Haouz, A., Forest, E. et al. (2021) Integrative study of the structural and dynamical properties of a KirBac3.1 mutant: functional implication of a highly conserved tryptophan in the transmembrane domain. *Int. J. Mol. Sci.* **23**, 335 https://doi.org/10.3390/ijms23010335

110 Jin, R., He, S., Black, K.A., Clarke, O.B., Wu, D., Bolla, J.R., et al. (2022) Ion currents through Kir potassium channels are gated by anionic lipids. *Nat. Commun*. **13**, 490 https://doi.org/10.1038/s41467-022-28148-4

111 Lee, S.J., Ren, F., Zangerl-Plessl, E.M., Heyman, S., Stary-Weinzinger, A., Yuan, P. et al. (2016) Structural basis of control of inward rectifier Kir2 channel gating by bulk anionic phospholipids. *J. Gen. Physiol.* **148**, 227237 https://doi.org/10.1085/jgp.201611616

112 Nishida, M. and MacKinnon, R. (2002) Structural basis of inward rectification: cytoplasmic pore of the G protein-gated inward rectifier GIRK1 at 1.8 Å resolution. *Cell* **111**, 957-965 https://doi.org/10.1016/s0092-8674(02)01227-8

113 Nishida, M., Cadene, M., Chait, B. T. and MacKinnon, R. (2007) Crystal structure of a Kir3.1-prokaryotic Kir channel chimera. *EMBO J.* **26**, 4005-4015 https://doi.org/10.1038/sj.emboj.7601828

114 Zhou, X., Zhao, C., Xu, H., Xu, Y., Zhan, L., Wang, P. et al. (2024) Pharmacological inhibition of Kir4.1 evokes rapid-onset antidepressant responses. *Nat. Chem. Biol.* **20**, 857-866 https://doi.org/10.1038/s41589-024-01555-y

115 Martin, G.M., Kandasamy, B., DiMaio, F., Yoshioka, C. and Shyng, S.L. (2017) Anti-diabetic drug binding site in a mammalian K_{ATP} channel revealed by Cryo-EM. *eLife* **6**, e31054 https://doi.org/10.7554/eLife.31054

116 Ding, D., Wang, M., Wu, J. X., Kang, Y. and Chen, L. (2019) The structural basis for the binding of repaglinide to the pancreatic K_{ATP} channel *Cell Rep.* **27**, 1848-1857 https://doi.org/10.1016/j.celrep.2019.04.050

117 Wang, M., Wu, J.X., Ding, D. and Chen, L. (2022) Structural insights into the mechanism of pancreatic K_{ATP} channel regulation by nucleotides. *Nat. Commun.* **13**, 2770 https://doi.org/10.1038/s41467-022-30430-4

118 Lee, K.P.K., Chen, J. and MacKinnon, R. (2017) Molecular structure of human KATP in complex with ATP and ADP. *eLife* **6**, e32481. https://doi.org/10.7554/eLife.32481

119 Sung, M.W., Yang, Z., Driggers, C.M., Patton, B.L., Mostofian, B., Russo, J.D. et al. (2021) Vascular K_{ATP} channel structural dynamics reveal regulatory mechanism by Mg-nucleotides. *Proc. Natl. Acad. Sci. U.S.A.*, **118**, e2109441118 https://doi.org/10.1073/pnas.2109441118
